# Lossless integration of multiple electronic health records for identifying pleiotropy using summary statistics

**DOI:** 10.1038/s41467-020-20211-2

**Published:** 2021-01-08

**Authors:** Ruowang Li, Rui Duan, Xinyuan Zhang, Thomas Lumley, Sarah Pendergrass, Christopher Bauer, Hakon Hakonarson, David S. Carrell, Jordan W. Smoller, Wei-Qi Wei, Robert Carroll, Digna R. Velez Edwards, Georgia Wiesner, Patrick Sleiman, Josh C. Denny, Jonathan D. Mosley, Marylyn D. Ritchie, Yong Chen, Jason H. Moore

**Affiliations:** 1 Department of Biostatistics, Epidemiology & Informatics, University of Pennsylvania, Philadelphia, PA USA; 2 Department of Biostatistics, Harvard T.H. Chan School of Public Health, Boston, MA USA; 3 Department of Statistics, University of Auckland, Auckland, New Zealand; 4 Biomedical and Translational Informatics Institute, Geisinger, Danville, PA USA; 5 Center for Applied Genomics, Children’s Hospital of Philadelphia, Philadelphia, PA USA; 6 Kaiser Permanente Washington Health Research Institute, Seattle, WA USA; 7 Psychiatric and Neurodevelopmental Genetics Unit, Center for Genomic Medicine, Massachusetts General Hospital, Boston, MA USA; 8 Department of Biomedical Informatics, Vanderbilt University Medical Centre, Nashville, TN USA; 9 Clinical and Translational Hereditary Cancer Program, Division of Genetic Medicine, Department of Medicine, Vanderbilt-Ingram Cancer Center, Vanderbilt University, Nashville, TN USA; 10 Department of Genetics, Perelman School of Medicine, University of Pennsylvania, Philadelphia, PA USA

**Keywords:** Data integration, Statistical methods

## Abstract

Increasingly, clinical phenotypes with matched genetic data from bio-bank linked electronic health records (EHRs) have been used for pleiotropy analyses. Thus far, pleiotropy analysis using individual-level EHR data has been limited to data from one site. However, it is desirable to integrate EHR data from multiple sites to improve the detection power and generalizability of the results. Due to privacy concerns, individual-level patients’ data are not easily shared across institutions. As a result, we introduce Sum-Share, a method designed to efficiently integrate EHR and genetic data from multiple sites to perform pleiotropy analysis. Sum-Share requires only summary-level data and one round of communication from each site, yet it produces identical test statistics compared with that of pooled individual-level data. Consequently, Sum-Share can achieve lossless integration of multiple datasets. Using real EHR data from eMERGE, Sum-Share is able to identify 1734 potential pleiotropic SNPs for five cardiovascular diseases.

## Abstract (web summary)

Thus far, pleiotropy analysis using individual-level Electronic Health Records data has been limited to data from one site. Here, the authors introduce Sum-Share, a method designed to efficiently and losslessly integrate EHR and genetic data from multiple sites to perform pleiotropy analysis.

## Introduction

Personalized prevention and treatment of diseases require a comprehensive understanding of the underlying genetic etiology. So far, numerous population-level genetic studies have been carried out to understand the associations between genetics and diseases. Notably, genome-wide association studies (GWAS) have systematically identified thousands of genetic loci associated with various human traits and diseases^1,2^. However, not all of the disease-causing loci are strongly associated with the phenotype, and thus a better understanding of the genetic etiology underlying complex diseases requires complementary approaches^3,4^. For most complex diseases, the current paradigm is that genes do not act in isolation. A growing body of genetic research suggests genetic pleiotropy, where one genetic locus or gene influences many phenotypes, is ubiquitous in complex traits^5–7^. Compared with the individual genetic variant association, analysis of pleiotropy can not only provide additional insights into shared genetic mechanisms among seemingly unrelated phenotypes^8^, but these connections could also be used as therapeutic targets for drug repositioning. In addition, leveraging information from pleiotropy has been shown to boost the statistical power to detect genetic associations and the predictive power of genetic risk factors^9,10^.

Electronic Health Record (EHR) data have rapidly become a promising data source for conducting genetic research due to the growing availability of EHR linked genetic data^11^. EHRs uniquely offer a comprehensive set of patients’ disease diagnoses as well as clinical measurements, which, in combination with genetic data, enable investigation of the genotype and phenotype relationships of multiple traits^6^. Indeed, many studies have utilized EHRs with linked genetic data to carry out phenome-wide association studies (PheWAS) to examine pleiotropy^12–14^. In PheWAS studies, association analysis of one or more single nucleotide polymorphisms (SNPs) can be used to identify pleiotropic effects on multiple phenotypes. While PheWAS is conceptually straightforward, it does not model multiple phenotypes together in the model. As a result, PheWAS may have a reduced power to detect pleiotropy due to both computational cost and multiple testing penalties. To alleviate the multiple testing burden, several methods have been developed to jointly model multiple phenotypes to detect pleiotropy. However, many of the joint-model methods suffer from limitations such as requiring phenotypes to be continuous. They also carry a high computational cost and may lack proper covariate adjustments^15^.

Recently, EHRs with linked genetic data have become increasingly available under initiatives such as the Electronic Medical Records and Genomics (eMERGE)^16^ and the UK BioBank^17^. Typically, an EHR system covers a specific service region and thus is representative of the patient population of the region. Therefore, data in each EHR system is limited in size and is also influenced by disease prevalence and demographic composition^18^. As a result, research findings using data from one EHR may not be generalizable to the whole population or reproducible across different EHR systems. Thus, it would be advantageous to integrate data from multiple EHRs to obtain generalizable results for a larger population and to improve power by maximizing sample size. When patients’ individual-level data are freely shareable, genetic and clinical data from multiple EHRs can be combined to perform a gold standard pleiotropy analysis. However, due to identifiability and privacy concerns, patients’ genetic and clinical information is often heavily protected and rarely shared across different EHRs. A potential solution is to utilize summary statistics to transfer information across datasets. As an example, the use of GWAS summary statistics has allowed low-cost and privacy-preserving alternative access to individuals’ genetic data. Summary statistics have been used successfully to perform single variant association tests, gene-based tests, fine-mapping, analysis of pleiotropic effects^19^ as well as meta-analyses^20–22^ without accessing patient-level data. However, analyses using summary statistics may introduce potential bias due to differences in study populations^23,24^. On the contrary, lossless integration, where the analysis of multiple datasets produces identical results compared with that of the combined individual-level data, could avoid this type of bias. Thus, methods that enable lossless privacy-preserving information sharing across EHRs are critically needed.

In this study, we developed Sum-Share **(**SUMmary Statistics from multiple electronic HeAlth Records for plEiotropy) to detect pleiotropy. This method allows for flexible covariate adjustment for each phenotype, is computationally more efficient than traditional methods, and leads to mathematically identical results as compared to analyses of pooled patient-level data from different sites. Importantly, Sum-Share only relies on summary statistics from different sites.

Using simulations, we show that Sum-Share is computationally efficient and achieved better statistical power than PheWAS in detecting pleiotropic effects. We apply Sum-Share to seven EHRs in eMERGE phase 3 data to detect pleiotropic effects between five cardiovascular-related phenotypes (obesity, hypothyroidism, type 2 diabetes, hypercholesterolemia, and hyperlipidemia). The integrated analysis identifies 1734 SNPs that showed significant pleiotropic associations compared with just 1 SNP when using EHR data from an individual site. To further evaluate our results, we re-analyze the significant SNPs using PheWAS in the UK BioBank data. This analysis identifies known genes as well as discovers new genes associated with cardiovascular diseases.

## Results

### Sum-Share

**Fig. 1:**
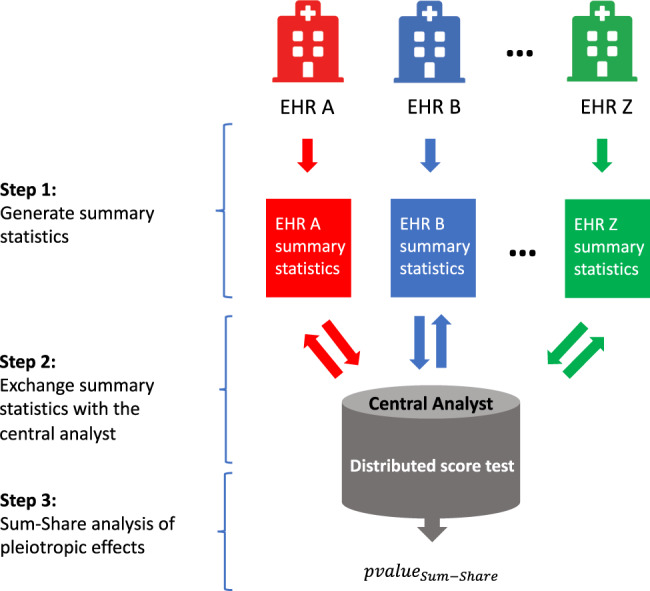
Schematic overview of the Sum-Share method. Sum-Share enables the investigation of pleiotropy using EHR data from multiple sites. The method involves three major steps. In step 1, each EHR will generate its own summary statistics, such as the mean or the covariance matrix. In step 2, the summary statistics from each EHR is transmitted to the central analyst and the analyst will generate pooled summary statistics and return them to each EHR. In step 3, each EHR will calculate test statistics using the pooled summary statistics and the individual test statistics will be integrated via the distributed score test to derive the final *p* value, denoted as *p* value_Sum-Share_.

Figure 1 provides an overview of the Sum-Share method. The goal of the method is to simultaneously identify pleiotropic effects between single SNPs and multiple phenotypes using data from multiple EHRs. The gold standard approach would be to pool individual-level patient data from multiple EHRs and perform pleiotropy tests on the combined data, known as individual patient-level data mega-analysis. However, this is rarely feasible in the real-world setting, as patient data are protected for privacy concerns and thus not easily shareable across EHRs. Sum-Share, which is based on the composite likelihood approach, decomposes the desired overall test statistics of the pleiotropic test into EHR specific test statistics. To obtain EHR specific test statistics, the method requires only summary-level information from each EHR. Importantly, the resulting p-value of the pleiotropic test from Sum-Share is identical to that of the gold-standard test (pooled data).

### Sum-Share is well powered to detect pleiotropy

**Fig. 2:**
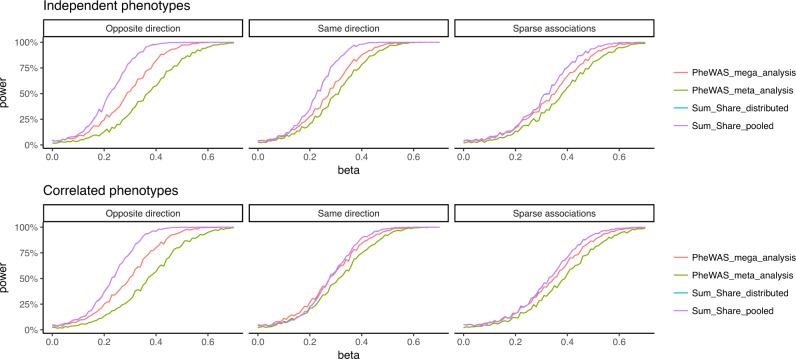
Power comparisons between Sum-Share and PheWAS. The phenotypes have ~40% prevalence and the SNP has a minor allele frequency of ~5%. The phenotypes and the SNP could have one of the three types of associations: opposite direction, same direction, and sparse associations. The phenotypes are independent (top panel) or correlated (bottom panel). Across beta values (association strength), power was compared between Sum-Share and PheWAS. Power was calculated as the percentage of times a method identified true significant associations out of 1000 repetitions. (Note: the blue line overlaps with the purple line).

To our knowledge, there are no methods that can losslessly perform pleiotropic tests using data from multiple EHRs without pooling individual-level data. Thus, our goal here is to demonstrate that Sum-Share improves statistical power as compared to standard PheWAS analysis under various simulation settings. To perform PheWAS, ten simulated EHR datasets were pooled together and standard PheWAS analysis was performed. The data pooling for PheWAS was achieved through meta-analysis or mega-analysis. In meta-analysis (PheWAS-meta), the summary statistics of each dataset were combined using the inverse-variance-weighting method. In contrast, mega-analysis (PheWAS- mega) pools the individual-level data and the analysis was performed on the larger pooled data. Sum-Share, as illustrated in Fig. 1, was also performed distributively using summary-level information from ten simulated datasets (Sum-Share-distributed). In addition, we also applied Sum-Share to the pooled individual-level data (Sum-Share-pooled) as a counterpart of the PheWAS-pooled analysis. The results show that Sum-Share achieved greater power than PheWAS in all settings, including scenarios in which SNPs associated with ten phenotypes under opposite direction effects, same direction effects, and sparse associations (Fig. 2). The performance comparisons also held when there were correlations among the phenotypes. Type 1 errors of the results were controlled at 5%. In addition, Sum-Share distributed and Sum-Share pooled have achieved identical power due to the lossless feature of the method. Additional simulations for common SNPs are included in Supplementary Fig. 1.

### Sum-Share can generate exact *p*-values using only summary-level data

**Fig. 3:**
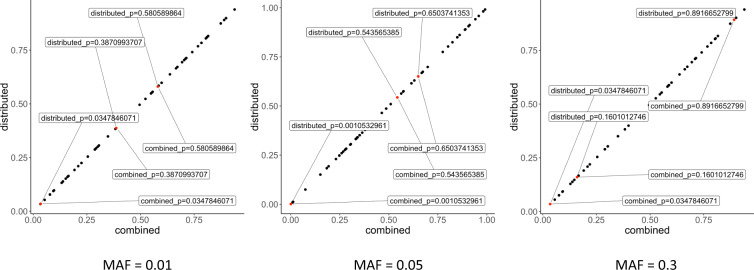
Correlation plot of p-values between Sum-Share and gold-standard analysis. SNPs and phenotypes were randomly selected from the eMERGE data to assess their associations. The SNPs were grouped into three categories according to their minor allele frequency (MAF = 0.01, 0.05, or 0.3) corresponding to left, middle, and right panels. *P*-values from the associations between each SNP and all phenotypes were obtained from either the distributed analysis (Sum-Share) or from the pooled individual-level data analysis (combined). Three SNPs in each category were randomly selected to display the corresponding *p*-values (up to the 10th significant digits).

As section ‘The Sum-Share algorithm’ and derivations in the Supplementary Note show, Sum-Share produces the same test statistics using summary statistics from multiple EHRs compared with pooled data. To further validate this lossless property using real data, randomly selected SNPs were analyzed for associations with five cardiovascular phenotypes in six EHRs. The SNPs were categorized by their minor allele frequencies (MAF) into common (MAF ≈ 0.3), low frequency (MAF ≈ 0.05) and rare (MAF ≈ 0.01) groups. *P*-values obtained from Sum-Share showed perfect correlation with *p*-values using the pooled data for all SNPs’ categories (Fig. 3).

### Detecting pleiotropic effects in cardiovascular traits across multiple EHRs

**Fig. 4:**
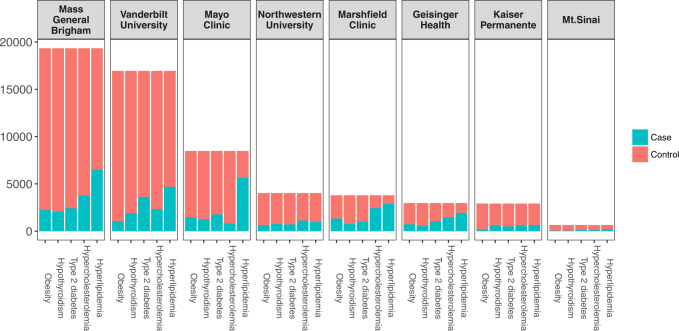
Disease prevalence of cardiovascular traits in eight EHRs. The patients’ case statuses were obtained using the following ICD-9 diagnosis codes: Obesity (ICD-9 278.00), Hypothyroidism (ICD-9 244.9), Type 2 diabetes (ICD-9 250.00), Hypercholesterolemia (ICD-9 272.0), and Hyperlipidemia (ICD-9 272.4). Each panel displays the disease prevalence for an EHR.

Genetic-linked EHR data from eight geographically distinct sites were used to detect pleiotropic effects among five common cardiovascular-related diseases (obesity, hypothyroidism, type 2 diabetes, hypercholesterolemia, and hyperlipidemia). Patients’ individual-level data from the EHRs can be combined through the eMERGE network. However, for this analysis, we only analyzed the summary-level data from the EHRs to demonstrate the method’s performance. The patients’ disease status was determined by the counts of the respective ICD-9 codes. Figure 4 shows the prevalence of each disease in eight EHRs.

To minimize population stratification, Sum-Share was only applied to patients with European ancestry. For each SNP, Sum-Share was used to evaluate associations between the SNP and five cardiovascular diseases, adjusting for gender and age. Importantly, as diseases could have different ages of onsets, Sum-Share was able to adjust for different ages for each disease. A current limitation of Sum-Share is that it cannot adjust for continuous covariates while maintaining the lossless property. Thus, the principal components adjustment for ancestry was not included in the analysis (see “Discussion”). In total, ~6.1 million SNPs were analyzed and their *p*-values were Bonferroni adjusted to account for multiple testing. As for comparisons, the analysis was carried out using data from each individual site as well as combined data from two of the largest sites (Mass General Brigham and Vanderbilt University) or all eight sites. For site-specific analyses, Sum-Share did not need to aggregate information from other sites, thus no distributed analysis was carried out. The combined eight sites analysis resulted in 1734 significantly associated SNPs, and the two-site analysis yielded 171 significant pleiotropic compared to only one SNP across the site-specific analyses (Table 1).

**Table 1:** Significant SNPs identified from individual and combined EHR analyses.

EHRs	Sample size	Significant SNP associations
Combined analysis using Sum-Share	59,136	1734
Mass General Brigham + Vanderbilt University	36,272	171
Mass General Brigham	19,329	0
Vanderbilt University	16,943	0
Mayo Clinic	8485	0
Northwestern University	4033	0
Marshfield Clinic	3801	1
Geisinger Health	2974	0
Kaiser Permanente/UW	2921	0
Mt Sinai	650	0

Across 6.1 million SNPs, each SNP was evaluated for its association to the five phenotypes. A single *p*-value is returned for each SNP that determines its significance with all phenotypes. In the individual EHR analysis, each site was analyzed separately. In the combined EHR analysis, multiple sites’ summary-level data were jointly analyzed by Sum-Share.

**Fig. 5:**
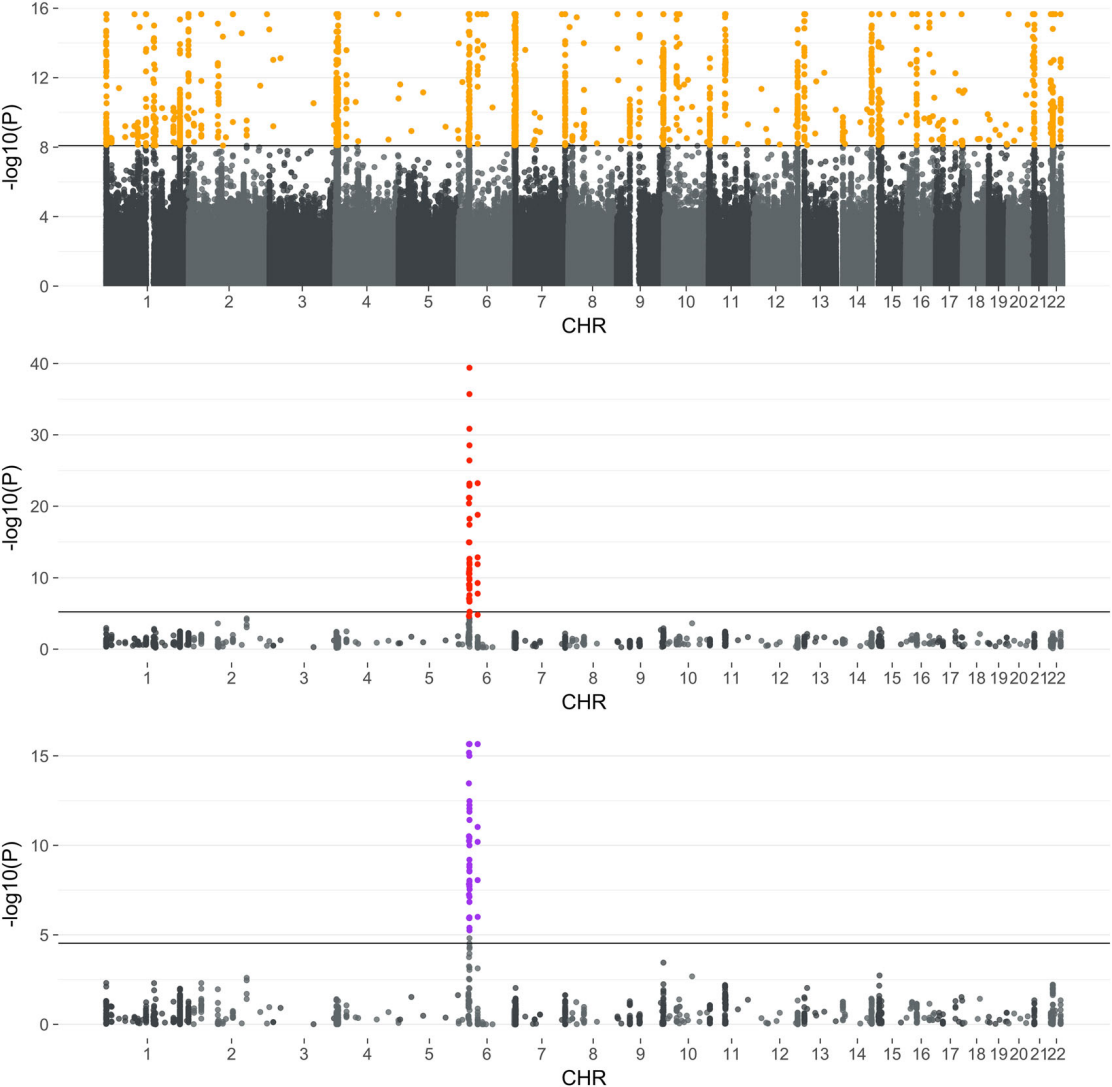
Manhattan plot of significant SNPs’ associations. The top Manhattan plot shows the association results of Sum-Share in eMERGE. Each *p*-value displayed (*y*-axis) is obtained from the test between an SNP (*x*-axis) and five phenotypes. The straight crossing line indicates the Bonferroni adjusted *p*-value threshold of 8.19 × 10^−9^. The middle Manhattan plot displays the significant SNPs identified in Sum-Share (orange dots in the top panel) analyzed using PheWAS in the UK BioBank data. Each *p*-value displayed (*y*-axis) is the minimum *p*-values between an SNP (*x*-axis) and five phenotypes. The straight-line indicates the Bonferroni adjusted *p*-value threshold of 0.05/(1698*5) = 5.89 × 10^−6^. The bottom Manhattan plot shows the same SNPs analyzed by Sum-Share in the UK BioBank data. The Bonferroni adjusted *p*-value threshold is 0.05/1698 = 2.94 × 10^−5^.

The 1734 significantly associated SNPs (*p* < 8.19 × 10^−9^) were displayed in Fig. 5. SNPs were mapped to 538 gene and gene transcripts using the Ensembl Variant Effect Predictor^25^ (Supplementary Data 1).

### Common SNPs identified between Sum-Share and PheWAS in UK BioBank data

Sum-Share and PheWAS differ in their approach in detecting pleiotropy associations. However, there can be commons SNPs that are identified by both approaches, which can indicate robust pleiotropic association of the SNPs. Thus, to further evaluate SNP associations from Sum-Share, significant SNPs identified in eMERGE data were re-analyzed using PheWAS analyses in the UK Biobank data. Out of 1734 significant SNPs found in the Sum-Share analysis of eight EHRs, 1698 SNPs were present in the UK BioBank dataset. Each SNP’s association with a disease was assessed using logistic regression while adjusting for gender and age. In total, 8490 associations were evaluated. 50 SNPs showed significant association (*p* < 5.89 × 10^−6^) with at least one of the phenotypes (Fig. 5). Many of the 50 SNPs were mapped to cardiovascular-related genes, including *BTNL2*, *FGFR3P1*, *HLA* family, *PRIM2*, and *RPL32P1* (Supplementary Data 2). Similarly, the same set of SNPs were re-analyzed using Sum-Share in the UK Biobank data. 54 SNPs showed significant associations (*p* < 2.94 × 10^−5^) (Supplementary Data 3). Out of the union of significant SNPs identified in the UK Biobank, 49 SNPs were shared by the two methods.

## Discussion

As many institutions and health systems have begun to utilize healthcare data, such as bio-bank linked EHR data, for research, integrating and exchanging information from multiple sites has emerged as a way to achieve more generalizable and robust research results. However, due to data privacy concerns, individual-level patients’ data are generally protected from cross-site transfers. As a result, we developed the method, Sum-Share, which can achieve lossless integration of summary statistics across multiple EHRs, while preserving the privacy of patients’ data.

Sum-Share achieves lossless integration of summary statistics through use of the composite likelihood method. As section ‘The Sum-Share algorithm’ shows, Sum-Share decomposes the likelihood function into summary-level statistics that can be calculated at each site. Each site then transfers the summary-level statistics to the central analyst to calculate the overall likelihood. To demonstrate the effectiveness of Sum-Share, we first conducted simulation studies to compare the method’s power with the widely used PheWAS method in detecting pleiotropic effects. We simulated known pleiotropic signals under different direction of effects, the strength of effect sizes, and phenotype correlations. The simulation results showed that Sum-Share achieved greater power than PheWAS in all settings (Fig. 2). Notably, our simulation favored PheWAS by allowing it to pool individual-level data from multiple EHRs, while our method only used summary-level data from these sites. Similarly, other multivariate methods to detect pleiotropy, such as MultiPhen^26^ and TATES^27^, also require individual-level data and thus were not compared. Next, we showed Sum-Share can losslessly integrate summary-level data by comparing the *p*-values obtained using summary-level data versus using pooled individual-level data. Figure 3 shows that the two sets of *p*-values are identical, which indicates that Sum-Share did not lose information by only using summary-level data. These simulation results demonstrate that Sum-Share is well-powered to detect pleiotropic effects.

To investigate the potential pleiotropy between cardiovascular-related diseases, we applied Sum-Share to seven EHR sites from eMERGE to detect potential pleiotropic SNPs for five cardiovascular diseases. The prevalence for the five diseases varied across sites, but hyperlipidemia was the most frequent disease diagnosis for all EHRs (Fig. 4). We identified 1734 significantly associated SNPs when all EHRs were analyzed together and one significant SNP in Marshfield Clinic EHR when EHRs were analyzed separately. The increased number of significant associations could be potentially explained in several ways. First, the straightforward explanation is that the increase in total sample size from the integration of seven EHRs led to an increased power to detect signals. Using simulation study, we showed that Sum-Share has higher power to detect signals using the integrated dataset compared to using only individual datasets (Supplementary Fig. 2). Second, genotype allele frequencies are known to influence the power to detect genetic associations. Rare or low-frequency SNPs have a much lower power to be detected in the presence of true signals. However, if a SNP is rare in some of the datasets but common in the other datasets. An integrated dataset may stabilize the allele frequencies of the SNPs. As simulation has shown, Sum-Share can still achieve high power when a SNP is common in only a portion of the datasets (Supplementary Fig. 3). We further evaluated the 1734 SNPs in the UK BioBank data using the PheWAS approach with the goal of determining whether any of the significant SNPs could be independently identified using an analogous method. 50 SNPs showed significant PheWAS associations with the five phenotypes. These SNPs mapped to genes including *BTNL2*, *HLA* family, and *PRIM2*. The *BTNL2* gene has been implicated in cardiac sarcoidosis^28^ and type 1 diabetes^29^. The *HLA* family genes contain the most polymorphic genetic regions in humans. Genes in this family are associated with over 100 diseases such as type 1 diabetes and autoimmune diseases^30^. The *PRIM2* gene was identified by a large-scale GWAS study to be associated with coronary artery disease^31^. Applying the Sum-Share method to the same SNPs, 54 SNPs were found to be significant. In addition, 49 out of the 50 SNPs were also identified by Sum-Share. As these 49 SNPs were identified and validated by two different methods in multiple datasets, these results increase confidence that they have true genetic associations with multiple phenotypes. While the high number of overlapping SNPs between Sum-Share and PheWAS identified in the UK Biobank can increase the confidence about our proposed method as well as the validity of the results. The relative low number of significant SNPs identified overall in the UK Biobank can be attributed to several potential factors. First, the eMERGE data contains patients recruited from the health systems in the United States, while the UK BioBank is a national biobank in the United Kingdom. As a result, there are potential differences between the two data in terms of the study design, demographics, and others. For example, there are no participants with age over 75 in the UK Biobank data used in the analysis. In contrast, a large number of participants in the eMERGE data are in that age group. Second, the phenotypes in the eMERGE data were derived from the ICD-9 diagnosis code, while ICD-10 codes were used in the UK Biobank data. While we used the closest matching codes in the two data, the codes were not completely the interchangeable. Finally, the quality of the corresponding significant SNPs in the UK Biobank data was low. As Supplementary Fig. 5 shows, almost half of the SNPs have more than 5% missing rate, a common threshold for genotyping quality.

Although we investigated genetic pleiotropy using multiple EHRs, the Sum-Share method can be generalized to other datasets, where there are one or more outcomes and covariates. Nevertheless, the method has several current limitations that require further development and evaluation. To preserve the lossless feature of Sum-Share, the method is only designed to analyze categorical (including binary) outcomes and covariates. Thus, it cannot adjust for continuous covariates such as principal components in the pleiotropic analysis. We compared the power of PheWAS with continuous covariates adjustment versus Sum-Share with categorical covariates (discretized continuous covariates). Sum-Share still has significantly higher power than PheWAS (Supplementary Fig. 4). In addition, the type 1 error was still maintained at 5%. In our analysis, we only analyzed unrelated patients of European descent to minimize effects from population stratification. We did, however, adjust for a different age for each phenotype in the same model. We believe this adjustment is important for analyses of pleiotropy, because different phenotypes may have different ages of onset. To our knowledge, no current multivariate method for pleiotropy analysis can adjust for multiple age variables. In order to adjust for continuous covariates, iterative distributed algorithms such as GLORE have been proposed^32^. However, the requirement of iterative communication of summary statistics is less feasible in our biobank data setting. Other recently proposed communication efficient distributed algorithms such as ODAL could be applied to adjust for continuous covariates at the price of yielding a slightly different estimate, compared to the pooled data base analysis^33,34^. Sum-Share also assumes homogeneous effects across datasets (Eq. 1). For genetic effects, we believe this assumption is justifiable when multiple datasets consist of samples with the same ethnic population because of the similar underlying biological mechanism. For other covariates, such as age and gender, there could be heterogeneous effects among different datasets. However, under the hypothesis testing framework, assuming homogeneous effects when heterogeneity exists is still useful because we can still detect an averaged effect^35^. In addition, while Sum-Share aims to preserve patient’s privacy by utilizing summary statistics of the dataset, there is still risk of identifiability with summary statistics and other publicly available genetic information^36–40^. This is especially problematic in smaller datasets or for rare events, where the summary statistics are not fully protective against privacy. However, the current practice of sharing patients’ data is that the data are shared within an established consortium or between known collaborators. Thus, Sum-Share would have a significantly reduced risk of exposing the patients’ privacy.

## Methods

### eMERGE EHR data

In this study, genotype data with linked EHR data was obtained from the eMERGE network^41^. Phase III of eMERGE includes 83,717 genotyped patients from 11 sites. The eight adult sites were included in the study: Marshfield Clinic Research Foundation, Vanderbilt University Medical Center, Kaiser Permanente Washington/University of Washington, Mayo Clinic, Northwestern University, Geisinger, Mt.Sinai, and Mass General Brigham (formerly Partners Healthcare). SNPs were imputed using the Haplotype Reference Consortium 1.1 reference under genome build 37, which resulted in 39 million total genetic variants^42^. SNP genotypes were filtered and processed using the standard pipeline^43^ so that the genotype and sample call rate were ≥99%, imputation score > 0.4, Hardy-Weinberg equilibrium *p*-value > 0.00001, and the MAF of the SNPs were ≥0.05. To reduce the effect of population structures, only unrelated individuals of European ancestry were used. For related individuals (π-hat ≥ 0.25, identity-by-descent), one of each pair was removed. In total, 59,136 individuals and 6,106,952 SNPs were analyzed.

### UK BioBank data

The UK Biobank publicly released phase 2 of deep genetic and phenotypic data on ~500,000 individuals across the United Kingdom^17^. Individuals were genotyped on two related types of genotype arrays (UK BiLEVE Axiom Array or UK Biobank Axiom Array) across 106 batches and imputed using the merged UK10K and 1000 Genomes phase 3 reference panels^44^. The UK Biobank data were obtained under application # 32133.

For sample quality control, first, individuals with SNPs missing at a rate >5% and high heterozygosity were removed due to poor quality. Second, one person within each pair of related individuals was removed. The relatedness threshold was set at second-degree relatives, which corresponds to the identity by descent π-hat value ≥0.25. Third, individuals who had mismatched self-reported and genetic-inferred sex were not included in the study. Genetic variants with imputation info score <0.3 and MAF <0.01 were excluded. For genotype data, we extracted the 1734 significant SNPs identified by Sum-Share. Out of 1734 SNPs, 1698 SNPs were also genotyped by the UK BioBank and passed the above quality control.

Because eMERGE’s phenotype data were derived from ICD-9 codes and UK BioBank contains mostly ICD-10 codes, we manually curated the corresponding ICD-10 codes for the five cardiovascular phenotypes. ICD-10 codes were E66.9 for obesity, E03.9 for hypothyroidism, E11.9 for type 2 diabetes, E78.0 for hypercholesterolemia, and E78.5 for hyperlipidemia.

### The Sum-Share algorithm

Sum-Share jointly studies the association between one SNP (denoted by *X*) with multiple phenotypes (*Y*_1_,…*Y*_*q*_). For each phenotype we assume

$${\mathrm{log}}\left\{ {\Pr \left( {Y_{\mathrm{j}} = 1} \right)\left| X \right.} \right\} = \alpha _{\mathrm{j}} + \beta _{\mathrm{j}}X,$$

where *β* is the corresponding log odds ratio of the SNP. Denote *α* = (*α*_1_,…*α*_*q*_) and *β* = (*β*_1_,…*β*_*q*_). To simultaneously model multiple phenotypes in multiple EHRs, Sum-Share used an adapted composite likelihood approach. More specifically, it assumed *K* clinical sites, and the sample size in the *k*-th site was *n*_*k*_. For the *i*-th subject in the *k*-th site, $d_{ik} = ( {y_{1ik}, \ldots ,y_{qik},x_{ik}})$ was observed. If the patient-level data could be pooled together, the log composite likelihood function based on the combined data was expressed as the following:

1 $$L\left( {\alpha ,\beta } \right) = \mathop {\sum }\limits_{k = 1}^K \mathop {\sum }\limits_{i = 1}^{n_k} \mathop {\sum }\limits_{j = 1}^q \left[ {y_{{\mathrm{jik}}}\left( {\alpha _j + x_{{\mathrm{ik}}}\beta _{\mathrm{j}}} \right) - \log \left\{ {1 + \exp \left( {\alpha _{\mathrm{j}} + x_{{\mathrm{ik}}}\beta _{\mathrm{j}}} \right)} \right\}} \right].$$

To investigate whether a given SNP had a pleiotropic effect, we proposed to construct a score test, where we test *H*_0_:*β* = 0, against *H*_α_:*β* *≠* 0. Denote $y_{ik} = (y_{1ik}, \ldots ,y_{qik})$ to be a vector of all *q* phenotypes, $\bar{y} = (\sum_{k = 1}^K \sum _{i = 1}^{n_k} y_{1ik}/n, \ldots ,\sum_{k = 1}^K \sum_{i = 1}^{n_k} y_{qik}/n)$ to be the sample mean *y*_*ik*_ of the combined dataset across all sites, $\bar x = {\sum _{k = 1}^K} {\sum _{i = 1}^{n_k}} {x_{{1}{ik}}}/{n}$ to be the sample mean of the SNP. The score test statistic can be constructed as

2 $$T = SV^{ - 1}S^T,$$

where *S* is the score function which is obtained by taking the first derivative of the likelihood function in (1), and *V* is the estimated variance of *S*. Through some derivation, we have

$$S = \mathop {\sum }\limits_{k = 1}^K \mathop {\sum }\limits_{i = 1}^{n_k} (x_{ik}y_{ik} - x_{ik}\bar y),$$

and

$$V = \mathop {\sum }\limits_{k = 1}^K \mathop {\sum }\limits_{i = 1}^{n_k} (x_{ik} - \bar x)^2\left( {y_{ik} - \bar y} \right)\left( {y_{ik} - \bar y} \right)^T.$$

Under the null hypothesis (*H*_0_:*β* = 0), the test statistic follows a *χ*^2^ distribution with *q* degrees of freedom asymptotically. Therefore, the *p*-value of the test can be calculated as

3 $$p = 1 - {{\Psi }}_{\mathrm{q}}\left( T \right),$$

where ${{\Psi }}_{\mathrm{q}}( \cdot )$ is the cumulative distribution function (CDF) of the centered *χ*^2^ distribution with *q* degrees of freedom.

With aggregated information $\bar y$ and $\bar x$, the two components of the test statistic *T*, i.e., the *q*-dimensional score function *S* and the *q* × *q*-dimensional matrix *V* can all be calculated distributivity. Each site only needs to calculate and share

4 $$S_k = \mathop {\sum }\limits_{i = 1}^{n_k} (x_{ik}y_{ik} - x_{ik}\bar y)\,{\mathrm{and}}\,V_k = \mathop {\sum }\limits_{i = 1}^{n_k} (x_{ik} - \bar x)^2\left( {y_{ik} - \bar y} \right)\left( {y_{ik} - \bar y} \right)^T,$$

which are all summary-level information.

We can generalize this method to adjust for potential confounding factors such as gender and age (see Supplementary Note).

The pseudo-code for obtaining the test statistic *T* distributivity is provided in Box 1.

Box 1.  **Pseudo-code  of  the  Sum-Share  algorithm**

1. **In site** *k* = 1,…*K* **do**
2. Calculate and share $\bar y_k = \mathop {\sum }\limits_{i = 1}^{n_k} y_{ik}/n_k$, $\bar x_k = \mathop {\sum }\limits_{i = 1}^{n_k} x_{ik}/n_k$, and sample size *n*_*k*_
3. **end**
4. **for** *k* = 1,…*K* **do**
5. Obtain the overall mean $\bar y = \mathop {\sum }\limits_{k = 1}^K n_k\bar y_k/n$, and $\bar x = \mathop {\sum }\limits_{k = 1}^K n_k\bar x_k/n$
6. Calculate and share *S*_*k*_ and *V*_*k*_ using (4)
7. **end**
8. Calculates $$S = \mathop {\sum }\limits_{k = 1}^K S_k$$, and $$V = \mathop {\sum }\limits_{k = 1}^K V_k$$
9. Obtain the test statistic *T* by (2) and obtain the *p*-value by (3).

### PheWAS

In PheWAS analysis, a logistic regression is applied between an SNP and each phenotype to determine its association. Each *p*-value from the SNP-phenotype association was Bonferroni adjusted by the number of phenotypes to obtain an adjusted *p*-value. The minimum adjusted *p*-value of all phenotypes was used to determine the power of PheWAS^6^.

### Power comparison with PheWAS in multiple EHRs

Various pleiotropic models were simulated to compare Sum-Share with PheWAS. As Sum-Share is able to integrate summary-level information from multiple EHRs, ten datasets under each pleiotropic model were simulated to mimic ten EHRs. Two types of data integration were performed for PheWAS: meta-analysis and mega-analysis. For meta-analysis, PheWAS analysis was performed on each dataset and the resulting association coefficients were integrated using inverse-variance-weighting. In mega-analysis, individual-level data from the ten datasets were pooled together to create a combined dataset, which was used for a PheWAS analysis. Similarly, Sum-Share was used to analyze these datasets distributively, through the integration of summary statistics from the ten datasets, or in a pooled analysis, which used the combined individual-level data. The procedure was repeated 1000 times. Power was calculated as the percentage of times a method identified significant pleiotropic associations out of 1000 repetitions.

Pleiotropic effects were generated from the following logistic model.

5 $${\mathrm{log}}\left( {\frac{{P(Y = 1|X)}}{{1 - P(Y = 1|X)}}} \right) = \beta_0 + X\beta,$$

with *n* patients and *q* phenotypes, *Y* is a *n* × *q* matrix of phenotypes, *X* is an *n* × 1 vector of SNP ∈{0,1,2} and following the Hardy-Weinberg equilibrium, *β* is a 1 × *q* coefficient vector, and β_0_ is the *n* × *q* intercept matrix of constant numbers. The SNP was simulated either as common, with MAF = 0.3, or low frequency, with MAF = 0.05. Ten phenotypes were simulated with binary disease status *y*_*i*_ ∈ {0,1}^*q*^ for each individual *i*. For each EHR, we simulated *n* = 100 patients, thus there were a total of 1000 patients in ten EHRs.

An SNP was simulated to exhibit different pleiotropic patterns with the ten phenotypes including: same direction, opposite direction, and sparse effects.

#### Same direction of effects

Under this model, an SNP was simulated to be associated with the ten phenotypes under the same effect, i.e., $\beta = \left( {\beta _1 = \beta _2 = \ldots = \beta _{10}} \right)$ and β_0 _= −0.5.

#### Opposite direction of effects

Under this model, an SNP was simulated to be associated with the first five phenotypes under one effect and the other five phenotypes under the opposite direction of effect, i.e., $\beta = \left( {\beta _1 = \beta _2 = \beta _3 = \beta _4 = \beta _5 = - \beta _6 = - \beta _7 = - \beta _8 = - \beta _9 = - \beta _{10}} \right)$ and β_0 _= −0.5.

#### Sparse effects

Under the sparsity model, not all phenotypes were associated with the SNP. A decaying model was used to simulate the genotype and phenotype associations. The model was $\beta = 2^{ - i} \ast \beta _1\,{\mathrm{with}}\,i = 1 \ldots 10$, and *β*_0 _= −0.5. Intuitively, the SNP was strongly associated with the first phenotype, but gradually decreased its association with the other phenotypes.

#### Correlation between phenotypes

**Fig. 6:**
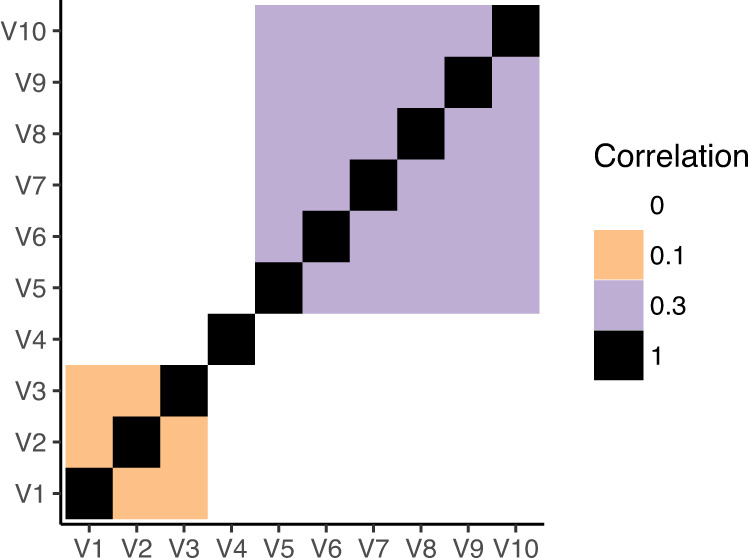
Correlation matrix for the correlated phenotypes. The phenotypes were simulated to exhibit two correlated clusters. The phenotypes within each cluster are correlated with each other. Phenotypes outside of the cluster are not correlated.

The above models assumed independence between the phenotypes. However, phenotypes could also be correlated while exhibiting pleiotropic associations. Thus, another set of simulation data was created that had the same pleiotropic effects, namely, same direction, opposite direction, and sparsity, at the same time the phenotypes were also made to be correlated. The correlation matrix for the phenotypes is shown in Fig. 6.

The rmvnorm function in the mvtnorm package was used to generate binary phenotypes from the correlation matrix^45^.

### The impact of sample size, allele frequency, and covariates adjustment on power

To evaluate the impact of sample size on the power of Sum-Share, three datasets with sample size *n* = 2000, 6000, and 12,000 were simulated as previously outlined (Section ‘Power comparison with PheWAS in multiple EHRs’, same direction of effects). Sum-Share was used to analyze the three data separately to obtain the power in each dataset. Then, Sum-Share was used to integrate the datasets to evaluate the power in the integrated dataset.

When integrating multiple genetic datasets, it is possible that the same SNP could have different allele frequencies across datasets, e.g., common in one dataset and rare in another. By integrating multiple datasets, an SNP will have an averaged allele frequency that is reflective of all data. The impact of the averaged allele frequency on power is evaluated as follows. Three equally sized (*n* = 2000) datasets were simulated as in Section ‘Power comparison with PheWAS in multiple EHRs’, same direction of effects. The MAFs of the SNP in the three datasets are: 0.05, 0.1, and 0.4. Sum-Share was used to integrate the three datasets and its power was evaluated. The power of Sum-Share using the integrated data was then compared to three additional datasets (*n* = 6000) that have the same underlying genetic model, but homogeneous in MAF = 0.05, 0.1, or 0.4. In summary, Sum-Share was used to compare the power using the integrated datasets (heterogenous in allele frequencies) and three individual datasets (homogeneous in allele frequency).

Due to the lossless characteristic of Sum-Share, it is currently not possible for Sum-Share to adjust for continuous covariates. As a result, the impact of continuous covariates adjustment is evaluated through the following simulation. The pleiotropic effects’ simulation was slightly modified based on Eq. (5)

6 $${\mathrm{log}}\left( {\frac{{P\left( {Y = 1{\mathrm{|}}X} \right)}}{{1 - P\left( {Y = 1{\mathrm{|}}X} \right)}}} \right) = \beta_0 + X\,*\,\beta+ {\mathrm{gender}}\,*\,\gamma + {\mathrm{age}}\,*\,\delta,$$

with gender_*i*_ ∈{0,1}^*q*^ generated from the Bernoulli distribution with *p* = 0.5 and ${\mathrm{age}}_i\sim {\mathrm{Normal}}({\mathrm{mean}} = 65,{\mathrm{sd}} = 20)$. In order to adjust for age, the continuous age was discretized as follows

$${\mathrm{age}}_{{\mathrm{discretized}}}\left\{ {\begin{array}{*{20}{c}} {{\mathrm{age}}{ \, <\,50,}}\hfill&0 \\ {{\mathrm{age}} {\ge 50}\,{\mathrm{and}}\,{\mathrm{age}} { \, <\,75,}}&1 \\ {{\mathrm{age}}{\ge\,75,}}\hfill& 2\end{array}} \right.$$

Ten datasets were generated using the “same direction of effects” model (Section ‘Power comparison with PheWAS in multiple EHRs’). The effects of gender and age were set as *β* = 0.1 and *δ* = 0.05, respectively. The value of *δ* was set so that it is equal to the average of the genetic effect size. Sum-Share was used to analyze the data adjusting for gender and age_discretized_. Similarly, PheWAS was used to analyze the same data adjusting for gender and the continuous age.

### *P*-values from Sum-Share and pooled analysis

To demonstrate that Sum-Share can produce the same *p*-values compared with the pooled EHR data (gold standard), we randomly selected sets of SNPs and phenotypes from six EHR sites (Table 2) to perform the comparison. To avoid bias due to MAF, rare (MAF ≈ 0.01, low frequency (MAF ≈  0.05) and common (MAF ≈ 0.3) SNPs were used in the comparison.

**Table 2:** Simulation using multiple eMERGE sites.

SNPs per set	50
MAF of SNPs in each set	0.01, 0.05, 0.3
Phenotypes	Malignant hypertension (ICD-9 401.0), Paroxysmal atrial tachycardia (ICD-9 427.0), Congestive heart failure (unspecified) (ICD-9 428.0), mitral valve disorder (ICD-9 424.0), Coronary atherosclerosis of unspecified type of vessel, native or graft (ICD-9 414.00)
eMERGE sites	Marshfield Clinic, Vanderbilt University, Kaiser Permanente/University of Washington Mayo Clinic, Northwestern University, Mass General Brigham

The phenotype definition from eMERGE was used^46,47^. Case status was determined by having ≥3 instances of the ICD-9 diagnosis code, and control status was determined by the absence of the ICD-9 code. Patients with one or two instances of the diagnosis codes were deemed as NA. In this analysis, NAs were imputed based on disease prevalence.

For each SNP, its association *p*-value with the five phenotypes was assessed using only summary statistics as implemented in Sum-Share or using the pooled patient-level data from all six sites.

### Application of Sum-Share to multiple EHRs

Adult patients with European ancestries in EHRs from Geisinger Health, Mass General Brigham, Kaiser Permanente, Marshfield Clinic, Mayo Clinic, Mt Sinai, Northwestern University, and Vanderbilt University were used. The prevalence of cardiovascular-related diseases was highest among the EHRs, thus five common cardiovascular diseases were selected to detect pleiotropic effects: obesity (ICD-9 278.00), hypothyroidism (ICD-9 244.9), Type 2 diabetes (ICD-9 250.00), Hypercholesterolemia (ICD-9 272.0), and Hyperlipidemia (ICD-9 272.4).

For each SNP, its association with the five phenotypes was evaluated using Sum-Share, adjusting for gender and age. For controls, age was determined as the age of patients’ last visit recorded in the EHR. For cases, each patient and disease diagnosis were associated with an age, which was calculated as the median age of the ICD-9 code assignments of a particular disease. The age was discretized into three nearly equal-sized bins: low (age < 50), medium (50 < age < 75), or high (age > 75). The discretization was necessary to preserve data privacy and to reduce computational cost. Without discretization, patients’ information could be exposed if, for example, only one patient at the age of 90 had a certain disease. Because the minimum p-value output by R was 2.22 × 10^−16^, *p*-values smaller than this threshold were reported as 2.22 × 10^−16^. This conversion did not affect the final results.

SNPs were deemed significant if they passed the Bonferroni adjusted *p*-value threshold 0.05/6106952 = 8.19 × 10^−9^. Significant SNPs were re-analyzed in the UK BioBank using PheWAS to identify any SNPs that can also be discovered using the standard approach. Similarly, the SNPs were also analyzed using the Sum-Share method. All of the significant SNPs as well as the subset of significant SNPs that were identified in the UK BioBank were annotated using the Ensembl Variant Effect Predictor to identify the corresponding genes^25^.

### Reporting summary

Further information on research design is available in the Nature Research Reporting Summary linked to this article.

## Supplementary information

Description of Additional Supplementary Files

Supplementary Information

Supplementary Data 1

Supplementary Data 2

Supplementary Data 3

Reporting Summary

## Data Availability

The eMERGE EHR data are not publicly accessible due to restricted user agreement. The UK biobank data is available through application (https://www.ukbiobank.ac.uk/). The UK biobank data used in this manuscript were obtained under application # 32133.

## Code availability

The code for Sum-Share is available on github (https://github.com/ruowangli/Sum-Share). The code is written in R.
