# Supplementary material for: Lossless integration of multiple electronic health records for identifying pleiotropy using summary statistics
Source: Nat Commun. 2021 Jan 8;12:168. doi: 10.1038/s41467-020-20211-2 (PMC7794298; doi:10.1038/s41467-020-20211-2)
Supplement: Supplementary file 1 — Description of Additional Supplementary Files [file 41467_2020_20211_MOESM1_ESM.pdf]

Title: Supplementary Data 1:
Description: Significantly associated SNPs identified by Sum-Share using the eMERGE data.

Title: Supplementary Data 2:
Description: Common SNPs identified by Sum-Share in eMERGE and PheWAS in the UK Biobank data.

Title: Supplementary Data 3:
Description: Common SNPs identified by Sum-Share in eMERGE and UK Biobank data
