# Supplementary material for: Lossless integration of multiple electronic health records for identifying pleiotropy using summary statistics
Source: Nat Commun. 2021 Jan 8;12:168. doi: 10.1038/s41467-020-20211-2 (PMC7794298; doi:10.1038/s41467-020-20211-2)
Supplement: Supplementary file 2 — Supplementary Information [file 41467_2020_20211_MOESM2_ESM.pdf]

Supplementary Figures and Notes for "Lossless integration of multiple electronic health records for identifying pleiotropy using summary statistics"

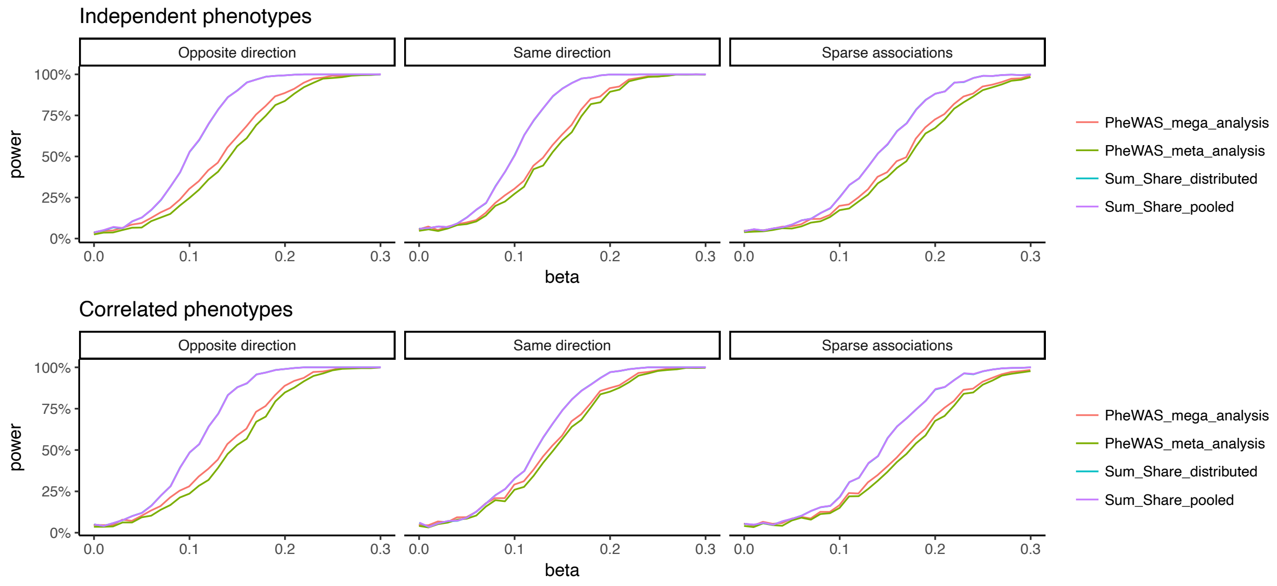

Supplemental Figure 1. Power comparisons between Sum-Share and PheWAS for common SNPs.

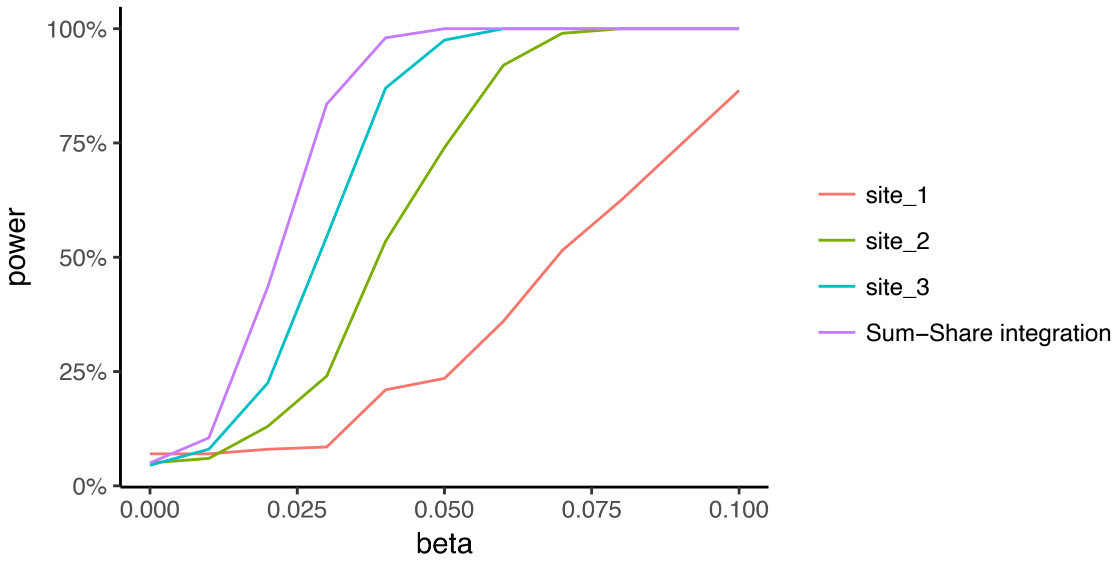

Supplemental Figure 2. Increased power through data integration

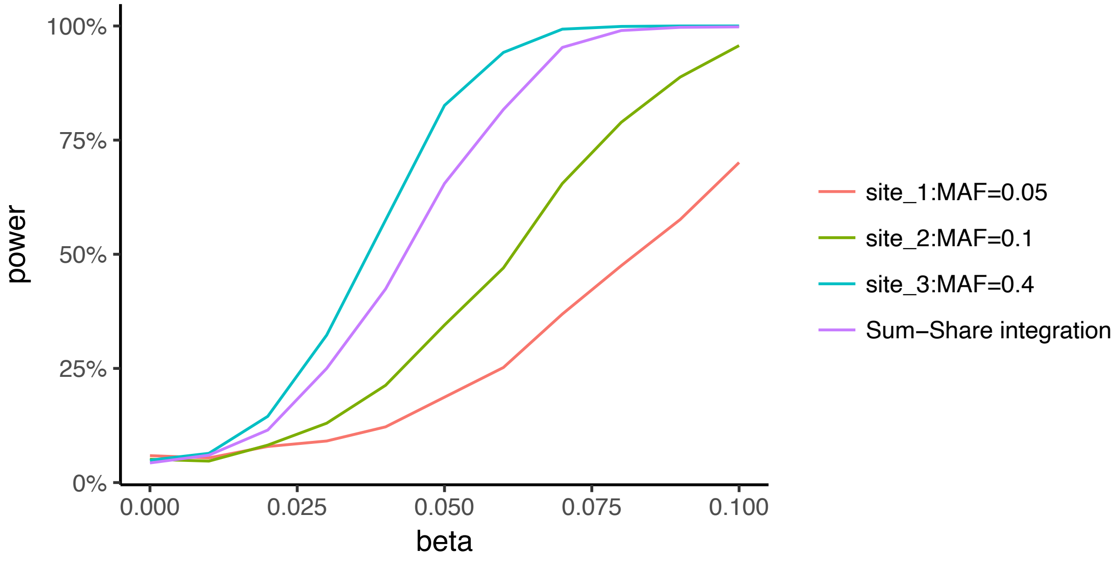

Supplemental Figure 3. The effects of integrating SNPs with heterogenous allele frequencies on power

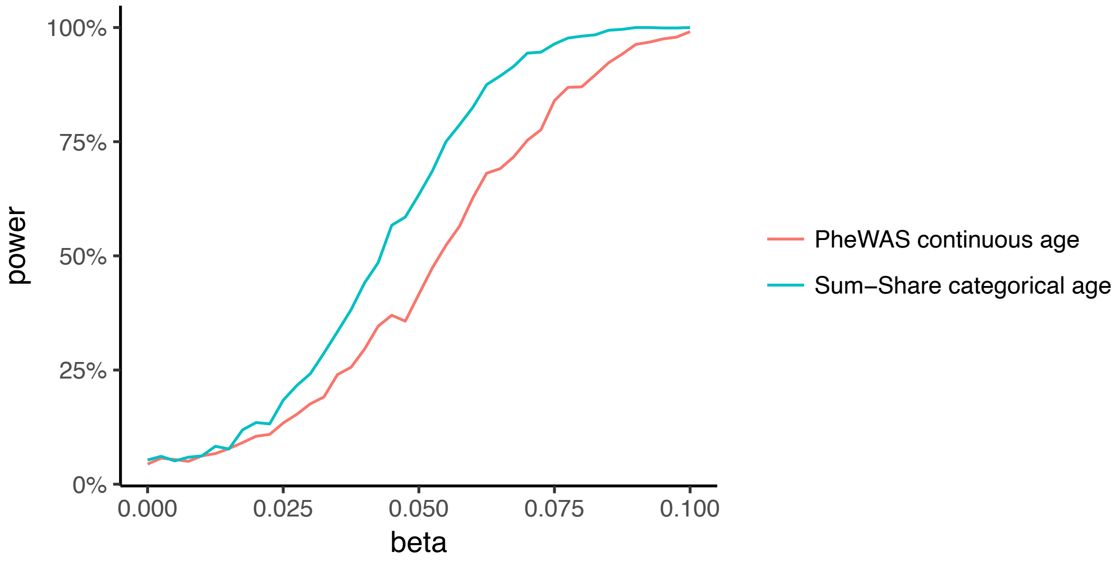

Supplemental Figure 4. Discretized continuous covariate adjustment's impact on Sum-Share

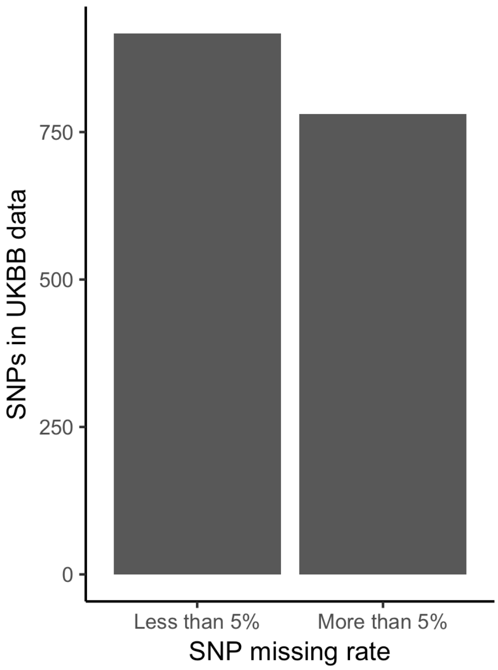

Supplemental Figure 5. Missing rate of SNPs used in the UK Biobank data.

Supplement Note for "Lossless integration of multiple electronic health records for identifying pleiotropy using summary statistics"

1. Sum-Share to adjust for potential confounding factors

We assume there are a set of categorical covariate variables, denoted by Z. Note that to protect privacy information, continuous variable can be discretized into groups to be included in the model. For example, we can adjust gender and age groups in the association analysis. For each outcome we assume

$$\log\{\Pr(Y_j = 1)|X, Z\} = \alpha_j + \beta_j X + \gamma_j^T Z,$$

where β is the corresponding log odds ratio of the SNP, adjusting for age and gender. Denote $\alpha = (\alpha_1, \ldots, \alpha_q)$, $\beta = (\beta_1, \ldots, \beta_q)$, and $\gamma = (\gamma_1, \ldots, \gamma_q)$.

For the i-th subject in the k-th site, we now observe $d_{ik} = (y_{1ik}, \ldots, y_{qik}, x_{ik}, z_{1ik}, z_{2ik})$. If the patient-level data can be pooled together, the log composite likelihood function based on the combined data can be expressed as the following:

$$\mathrm{L}(\theta) = \sum_{k=1}^{K} \sum_{i=1}^{n_k} l(\theta; d_{ik}) = \sum_{k=1}^{K} \sum_{i=1}^{n_k} \sum_{j=1}^{q} [y_{jik}(\alpha_j + x_{ik}\beta_j + z_{ik}^T\gamma_j) - \log\{1 + \exp(\alpha_j + x_{ik}\beta_j + z_{ik}^T\gamma_j)\}], \quad (1)$$

where $\theta = (\beta, \alpha, \gamma)$. To investigate whether the SNP has pleiotropic effect, we proposed to test $H_0: \beta = 0$, against $H_\alpha: \beta \neq 0$. Based on the log likelihood function in (1), we construct a score test statistic

$$T = SV^{-1}S^T \quad (2)$$

where S is the score function defined as

$$S = \sum_{k=1}^{K} \sum_{i=1}^{n_k} s_{ik} \quad (3)$$

with s_{ik} to be the q-dimensional score function of the i-th subject in the j-th site, defined as

$$s_{ik} = (\{y_{ji1} - expit\ (\hat{\alpha}_1 + z_{ik}^T\hat{\gamma}_1)\}x_{ik}, \ldots, \{y_{jiq} - expit\ (\hat{\alpha}_q + z_{ik}^T\hat{\gamma}_q)\}x_{ik}). \quad (4)$$

Let $\eta = (\alpha, \gamma)$, the term V in (2) is a $q \times q$ dimensional vector defined as

$$V = Q_{\beta\beta} - 2H_{\beta\eta}H_{\eta\eta}^{-1}Q_{\beta\eta}^T + H_{\beta\eta}H_{\eta\eta}^{-1}Q_{\eta\eta}H_{\eta\eta}^{-1}H_{\beta\eta}^T. \quad (5)$$

where $Q_{\beta\beta}, Q_{\beta\eta}, Q_{\eta\eta}$ are the corresponding submatrices of Q, defined as

$$Q = \sum_{k=1}^{K} \sum_{i=1}^{n_k} \frac{\partial l(0,\hat{\eta}; d_{ik})}{\partial\theta} \frac{\partial l(0,\hat{\eta}; d_{ik})}{\partial\theta}^T \quad (6)$$

and $H_{\beta\beta}, H_{\beta\eta}, H_{\eta\eta}$ are the corresponding submatrices of H, defined as

$$H = \sum_{k=1}^{K}\sum_{i=1}^{n_k}\frac{\partial^2 l(0,\hat{\eta};d_{ik})}{\partial\theta^2}. \quad (7)$$

The term $\hat{\eta}$ is estimated through a reduced model assuming $\beta = 0$, i.e.,

$$\hat{\eta}_j = (\hat{\alpha}_j, \hat{\gamma}_j) = argmax_{\alpha_j,\gamma_j}\sum_{k=1}^{K}\sum_{i=1}^{n_k}[y_{jik}(\alpha_j + z_{ik}^T\gamma_j) - \log\{1+\exp(\alpha_j + z_{ik}^T\gamma_j)\}]$$

The test statistic in (2) can be shown to follow a χ^2 distribution with q degrees of freedom asymptotically.

From Equations (2) - (7), we observe that given $(\hat{\alpha}, \hat{\gamma})$, each component of the test statistic, S, H and Q, can be distributively calculated by $S = \sum_{k=1}^{K} S_k$, $H = \sum_{k=1}^{K} H_k$,and $Q = \sum_{k=1}^{K} Q_k$, where the k-th site calculates and shares

$$S_k = \sum_{i=1}^{n_k} s_{ik}, H_k = \sum_{i=1}^{n_k}\frac{\partial^2 l(0,\hat{\eta};d_{ik})}{\partial\theta^2}, Q_k = \sum_{i=1}^{n_k}\frac{\partial l(0,\hat{\eta};d_{ik})}{\partial\theta}\frac{\partial l(0,\hat{\eta};d_{ik})}{\partial\theta}^T. \quad (8)$$

Since S_k, H_k and Q_k are all summation over individual-level quantities, they are summary-level statistics and no patient-level information is shared when sharing them.

To calculate the test statistic T in a distributed manner, we also have to obtain $\hat{\alpha}, \hat{\gamma}$. Since the all covariates in Z are categorial, the estimator $\hat{\alpha}, \hat{\gamma}$ can be obtained based on only the aggregated frequency table of Z, which does not contain patient-level information.

As an example, we assume Z includes gender and age, and age categorized into three groups. Let Z_1 denote gender, Z_2 and Z_3 denote the two dummy variables for age group setting the youngest group as the reference level, we have three covariates to adjust and the corresponding regression coefficients for the j-th outcome are $\gamma_j = (\gamma_{1j}, \gamma_{2j}, \gamma_{3j})$. Suppose we can get the following aggregated three-way frequency table for each outcome in combined dataset;

Table 1: three-way frequency table of the j-th outcome age and gender using the combined data.

$Y_j = 0$		Age 1 (reference)	Age 2	Age 3
	Male (reference)	m_{010}	m_{020}	m_{030}
	Female	m_{011}	m_{021}	m_{031}
$Y_j = 1$		Age 1	Age 2	Age 3
	Male (reference)	m_{110}	m_{120}	m_{130}
	Female	m_{111}	m_{121}	m_{131}

The parameter $(\hat{\alpha}_j, \hat{\gamma}_j)$ can also be estimated through the following optimization problem

$$\left(\hat{\alpha}_j, \hat{\gamma}_j\right) = argmax_{\alpha_j, \gamma_j}\ m_{110}\alpha_j + m_{111}(\alpha_j + \gamma_{1j}) + m_{120} + (m_{010} + m_{110})(\alpha_j + \gamma_{2j}) + m_{121}(\alpha_j + \gamma_{1j} + \gamma_{2j}) + m_{130}(\alpha_j + \gamma_{3j}) + m_{131}(\alpha_j + \gamma_{1j} + \gamma_{3j}) - \log\{1 + \exp \alpha_j\} - (m_{011} + m_{111}) \log\{1 + \exp(\alpha_j + \gamma_{1j})\} - (m_{020} + m_{120}) \log\{1 + \exp(\alpha_j + \gamma_{2j})\} - (m_{021} + m_{121}) \log\{1 + \exp(\alpha_j + \gamma_{1j} + \gamma_{2j})\} - (m_{030} + m_{130}) \log\{1 + \exp(\alpha_j + \gamma_{3j})\} - (m_{031} + m_{131})\ \log\{1 + \exp(\alpha_j + \gamma_{1j} + \gamma_{3j})\}. \quad (9)$$

The frequency table in Table 1 can be obtained by the frequency table from each site. As a result, the test statistic in (2) can be calculated distributivity without transferring patient-level information. The pseudo-code of the algorithm is shown below:

Algorithm 2. Pseudo-code of the Sum-Share algorithm with covariates

1. **for** $k = 1, \ldots K$ **do**
2. Calculate and share the frequency table based on data at the k-th site
3. **end**
4. **for** $k = 1, \ldots K$ **do**
5. Obtain the combined frequency table and obtain the estimates $(\hat{\alpha}, \hat{\gamma})$ using (9)
6. Calculate and share S_k and H_K and Q_K by (8)
7. **end**
8. Calculates S, H, and Q, and obtain V by (5)
9. Obtain the test statistic by (2).
